# Supplementary material for: Sex-Specific Electrocortical Interactions in a Color Recognition Task in Men and Women with Opioid Use Disorder
Source: Biomedicines. 2025 Dec 8;13(12):3002. doi: 10.3390/biomedicines13123002 (PMC12730701; doi:10.3390/biomedicines13123002)
Supplement: Supplementary file 1 [file biomedicines-13-03002-s001.zip › biomedicines-3957031-supplementary.pdf]

Table S1. *Neural and Physiological Substrates of Event-Related Potentials (ERP) and Associated Cognitive Processes*

ERP Components	Electrode Sites	Polarity (+ or −)	Time (msec) Start	Peak	Associated Neural Substrates	Cognitive Processing Stimuli and Other Factors
Visual						
C1	Posterior- midline	+ and − (varies)	40-60	80-100	V1 (primary visual cortex) – enfolded in calcarine fissure (+ upper field, - lower field)	First major visual component; highly sensitive to contrast and spatial frequency. Summates with P1 if + (horizontal stimuli midline).
P100 (P1)	Lateral Occipital	+	60-90	100-130	Early – dorsal extrastriate cortex (middle occipital gyrus). Later – ventral fusiform gyrus 30 distinct areas within 100ms	Variation in stimulus parameters: contrast, spatial frequency direction, subject state of arousal.
N100 (N1)	Anterior	−	75-100	2 @ 150-200	1. Parietal cortex 2. Lateral occipital cortex	Spatial attention; larger for discrimination than detection.
P200 (P2)	Anterior and Central	+	Follows N1		Hard to distinguish from overlapping N1, N2, P3	Larger for target and infrequent, simple stimulus.
N170 and Vertex Positive Potential	Vertex	−	150	170	Lateral occipital, right hemisphere; none in inferotemporal cortex	Attention; endogenous components.
N200 (N2)	Posterior	−	150	350	occipital	Spatially viewed *deviant* task—if task relevant; orienting reflex; breaks into 3 distinct components. [2] presents very young (1 month); discriminating, higher amplitude in sleep (most prominent at beginning of sleep), automatic and controlled attention; detect group differences.
N2a	Posterior	−	200	220		deviant task-irrelevant = mismatch negativity (MMN).
N2b	Posterior	−	200	220		Deviant task related; bilateral and probability sensitive - unsure if homologous to auditory N2b neural processing.
N2pc	Posterior – contralateral to location of target	−	200	220	Occipital and parietal (pc)	component seen posterior and contralateral (pc) to location of target; not probability sensitive; focus of spatial attention on target; seen in visual working memory tasks; working memory maintenance.

Visual and Auditory						
P300 (P3)	Frontal maximal	+	300	550	Not certain – no clear consensus of what neural or cognitive processes involved. [2]temporal lobectomies decrease P300; prefrontal lesions decrease AMP in fronto central; widespread neurons – hippocampi, frontal lobes, thalamus in humans; parieto-central/ centro-frontal changes with maturation	Affects AMP and LAT – AMP smaller if subject uncertain/ larger with increased difficulty; LAT increase if experiment influences processing; probability related; categorized mental task; influenced by unpredictable infrequent shift in tone pitch or intensity unexpected. [2]cognitive processing – not motivational; LAT: small decrease 4-10 yrs (550 to 474ms), significant drop 12 yrs (339 to 344ms), constant during perceptual and motor interference, increases when load increased(searching memory for more information; AMP: 10 yrs broad distributed scalp display/ adolescent sharp time phasic with maximum at parietal midline/ as get older to frontal—increased temporal separation smaller vertex to broader frontal position—from fronto-central to parieto-central occurs with age; high AMP reflects confidence in perceptual decision, increases if target improbable.
N400 (LN-late negative)	Central and parietal – right hemisphere	−	350	450	Left anterior medial temporal lobe (auditory)	Language specific. Response to semantics misuse in language –sentence doesn't match preceding lines.
P600 (LP-Late Positive)	Left frontal	−	300	700	Frontal cortex; left hemisphere activity dependent	Syntax of conversation, i.e., wh__? questions.
RP (Response time)	Depends on efforts used by subject	+ − +	100ms before response			Inattention – readiness potential; lateralized to the hand making the response.
LRP (lateralized response potential)	Contralateral to hand vs foot as they together work to do task	+			Motor cortex	Prepared for response; responses are faster when LRP is larger at moment of stimulus.
ERN (error related negativity)	Frontal central	−	After error made		Anterior cingulate cortex	Error detection using negative feedback from wrong response or by watching others make a mistake.
N10	Vertex potential Action potentials	− and +	10-20	20-100	Somatosensory, olfactory, and gustatory responses	These are not post-synaptic potentials but action potentials from peripheral nerves, followed by subcortical components and short and medium latency cortical components. N1 followed by P2 together

			are called vertex potential. Hard to record olfactory and gustatory ERP responses.

Note: AMP = amplitude; LAT = latency; yrs = years; N1 = N100; P1 = P100; N2 = N200; P2 = P200; P3 = P300.

[1]As summarized from Luck, S. J. [22].

[2]See Grant, M. L. [57]

[3]See Frith, C. D., & Friston, K. J. [58].
